# Supplementary figures and images for: Zoonotic Mutation of Highly Pathogenic Avian Influenza H5N1 Virus Identified in the Brain of Multiple Wild Carnivore Species
Source: Pathogens. 2023 Jan 20;12(2):168. doi: 10.3390/pathogens12020168 (PMC9961074; doi:10.3390/pathogens12020168)

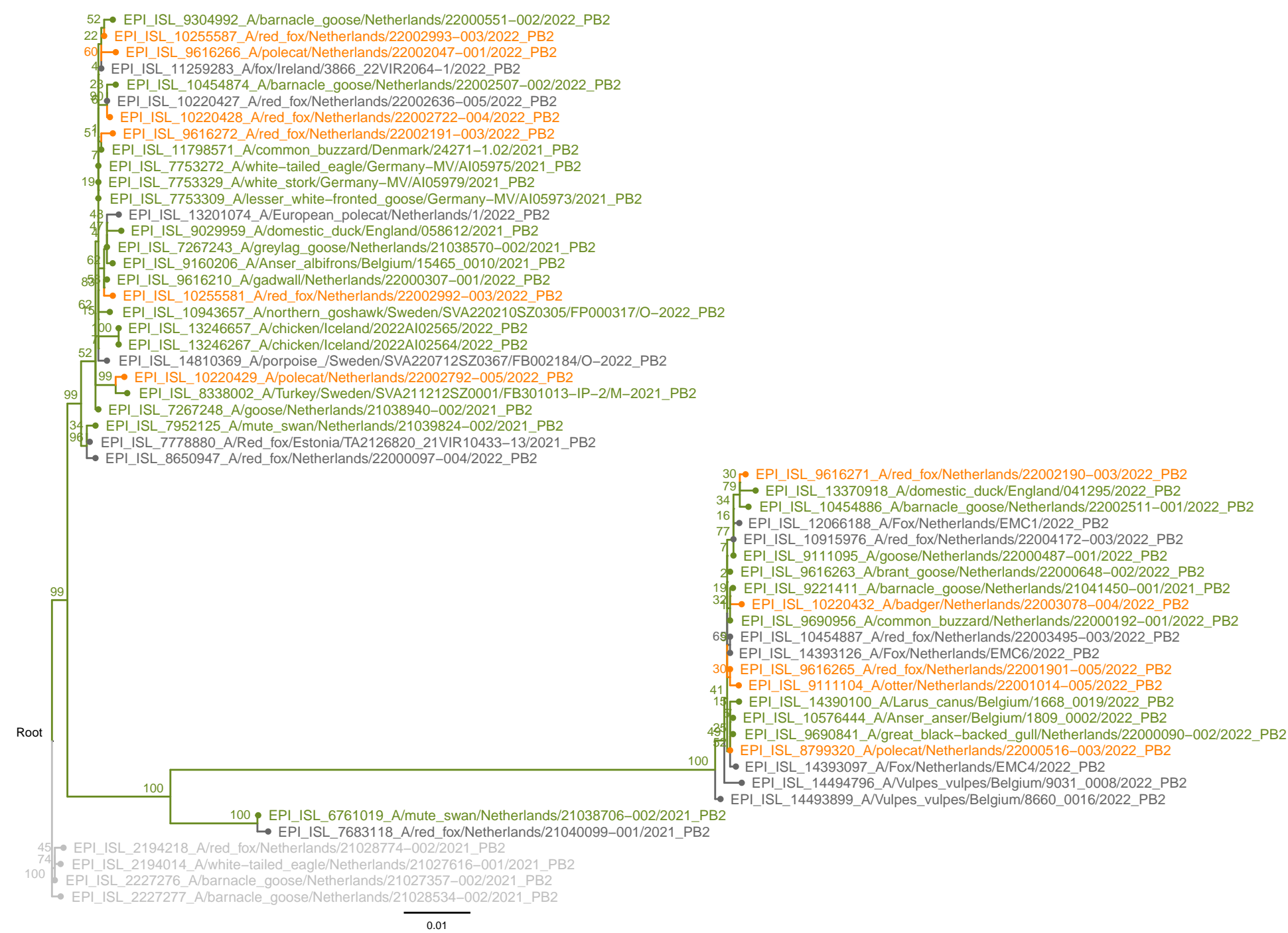

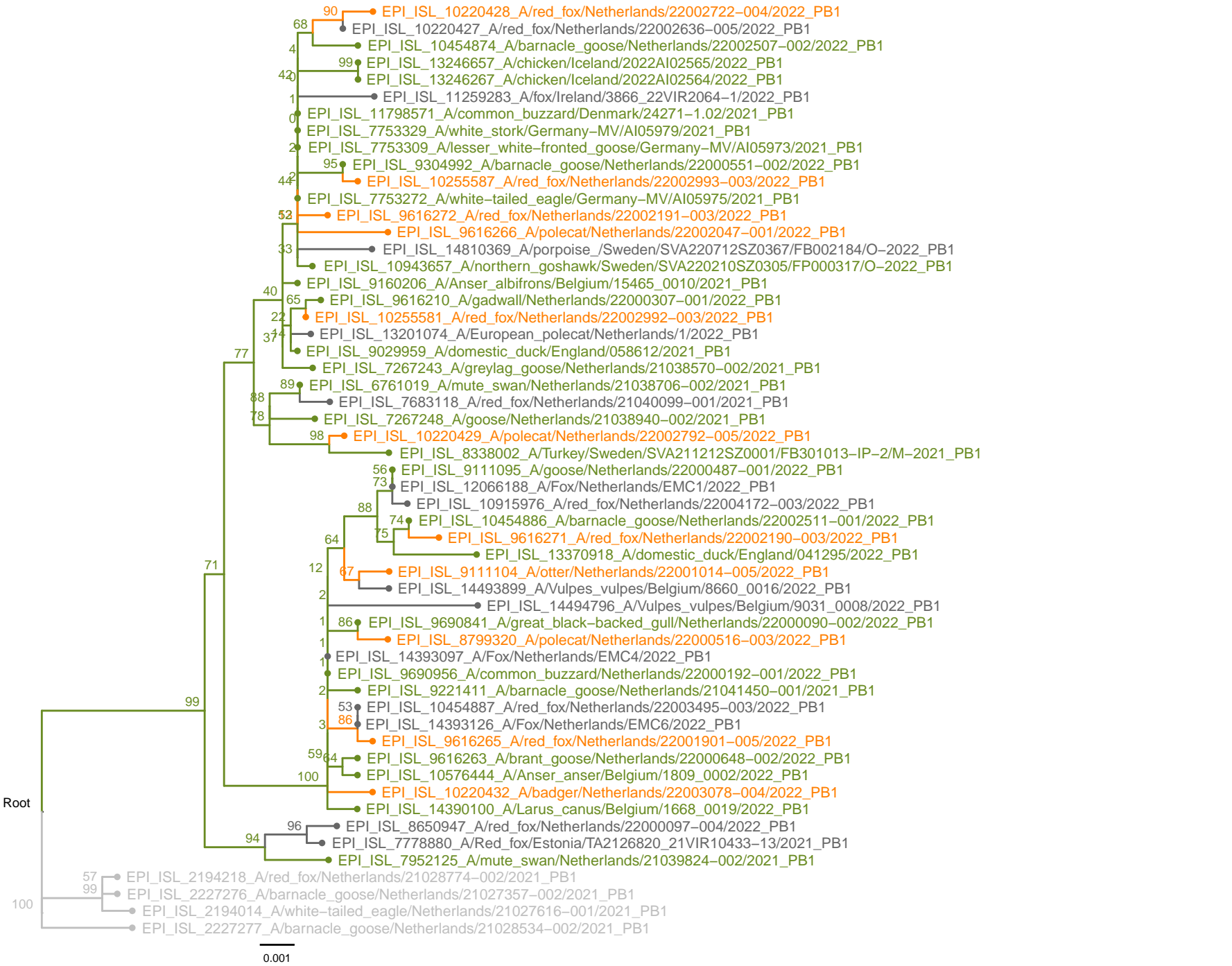

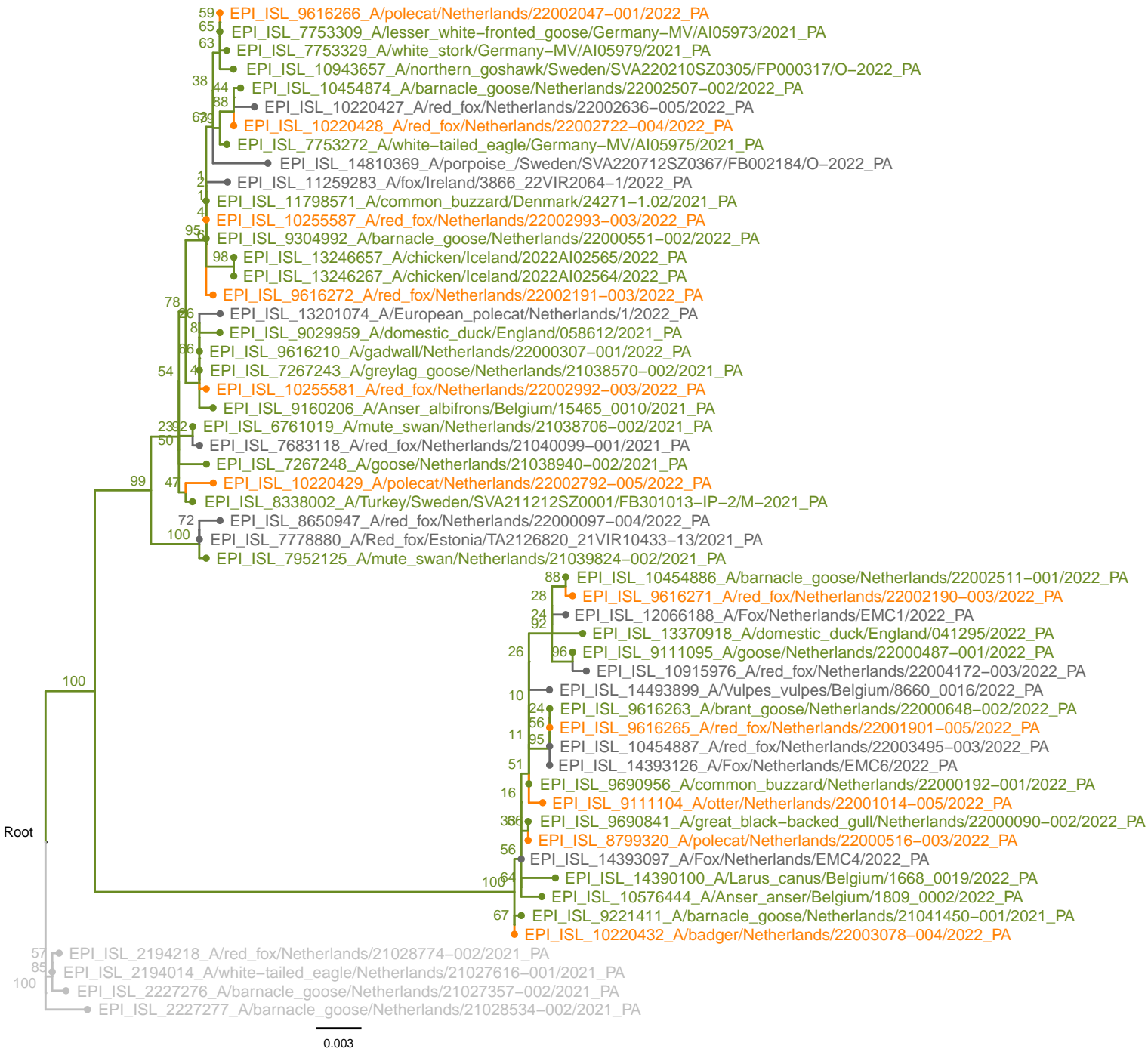

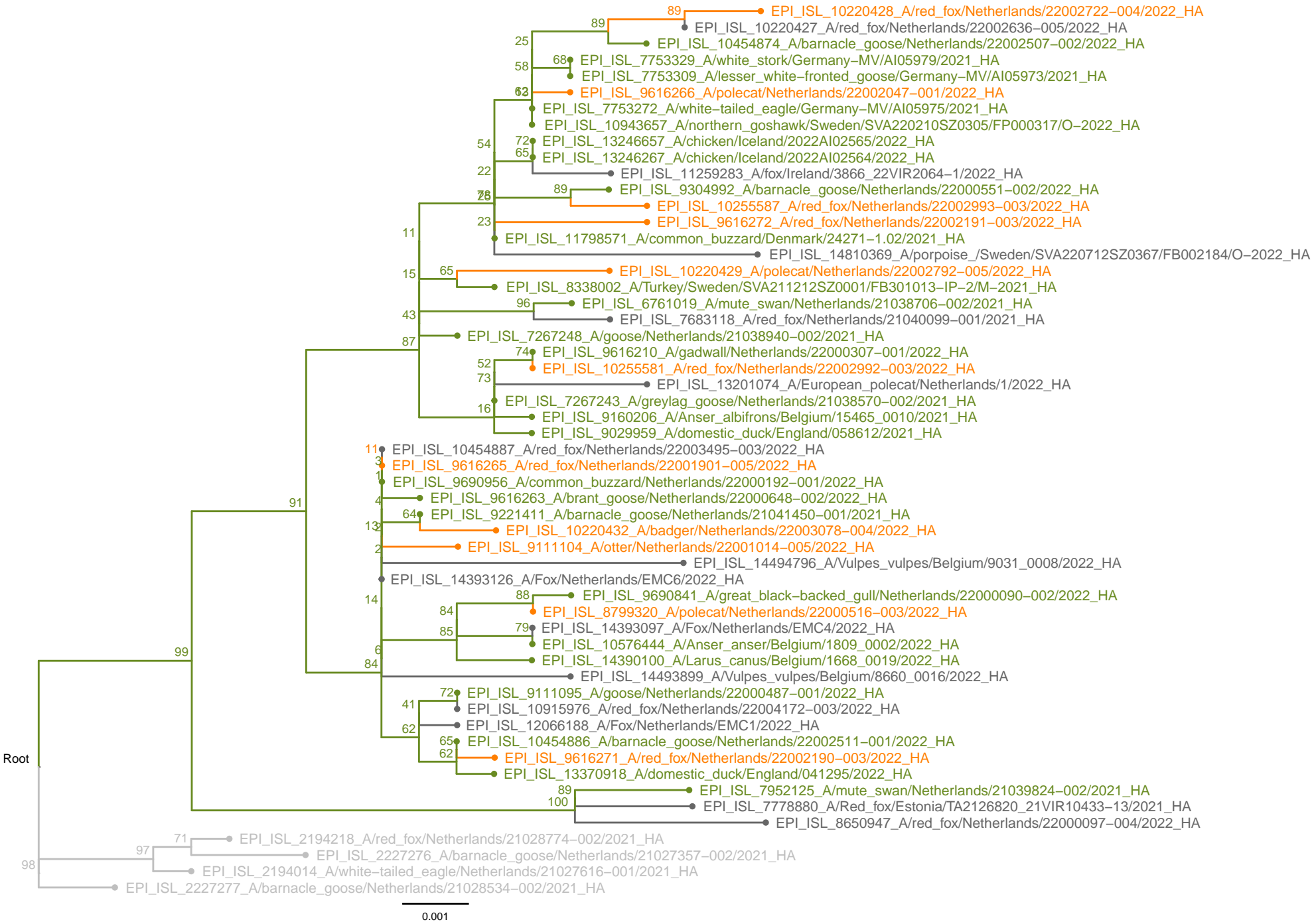

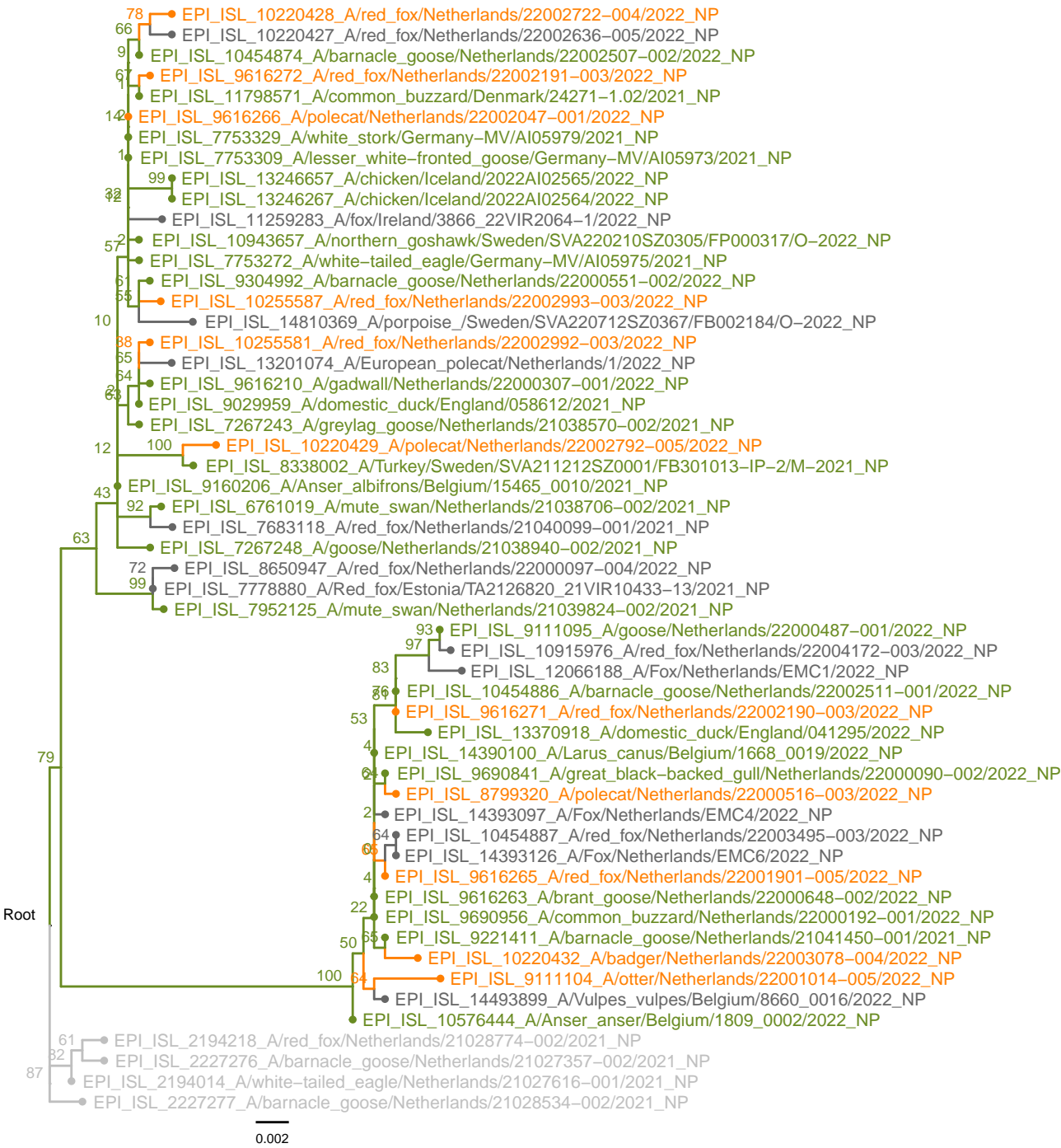

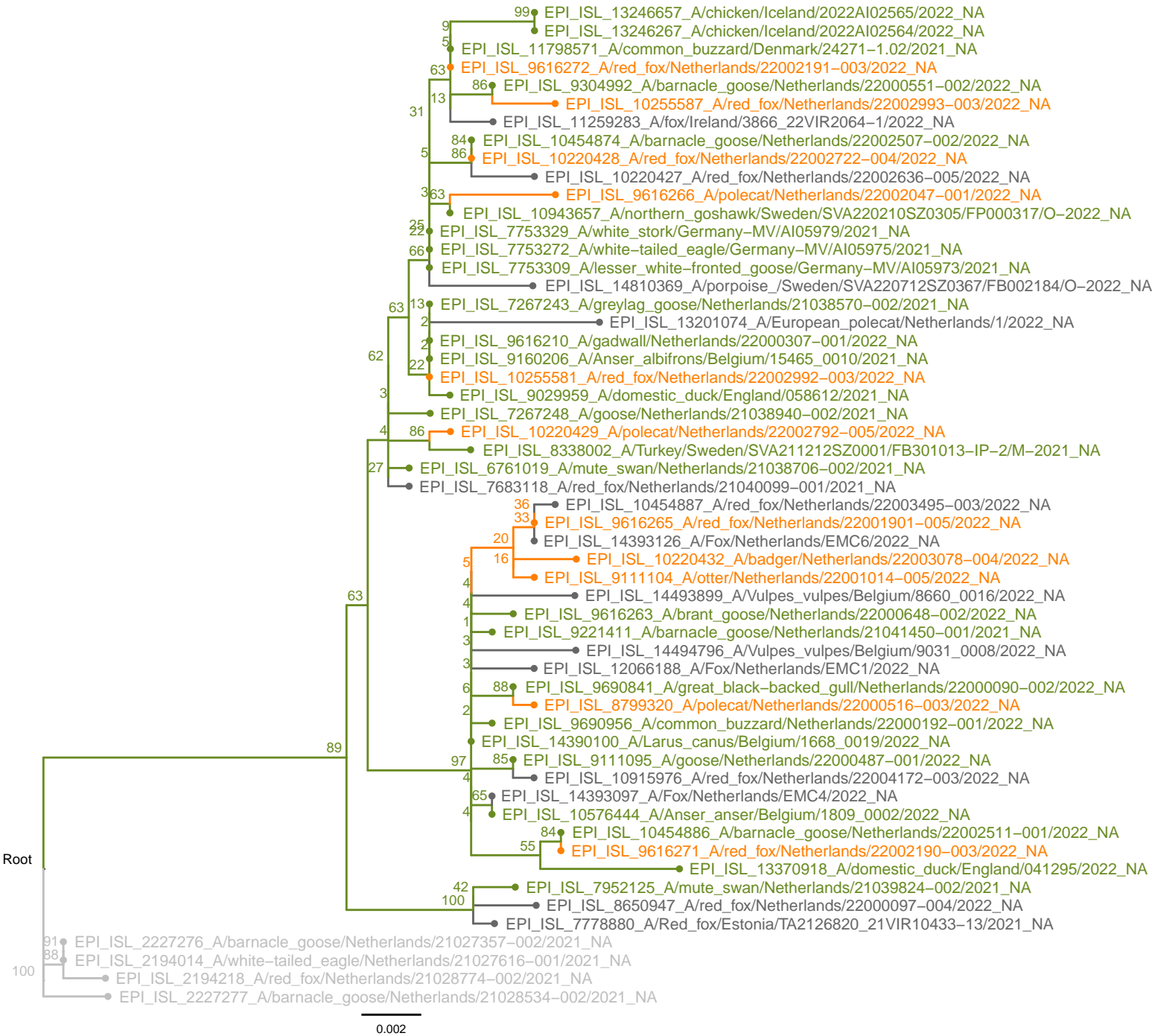

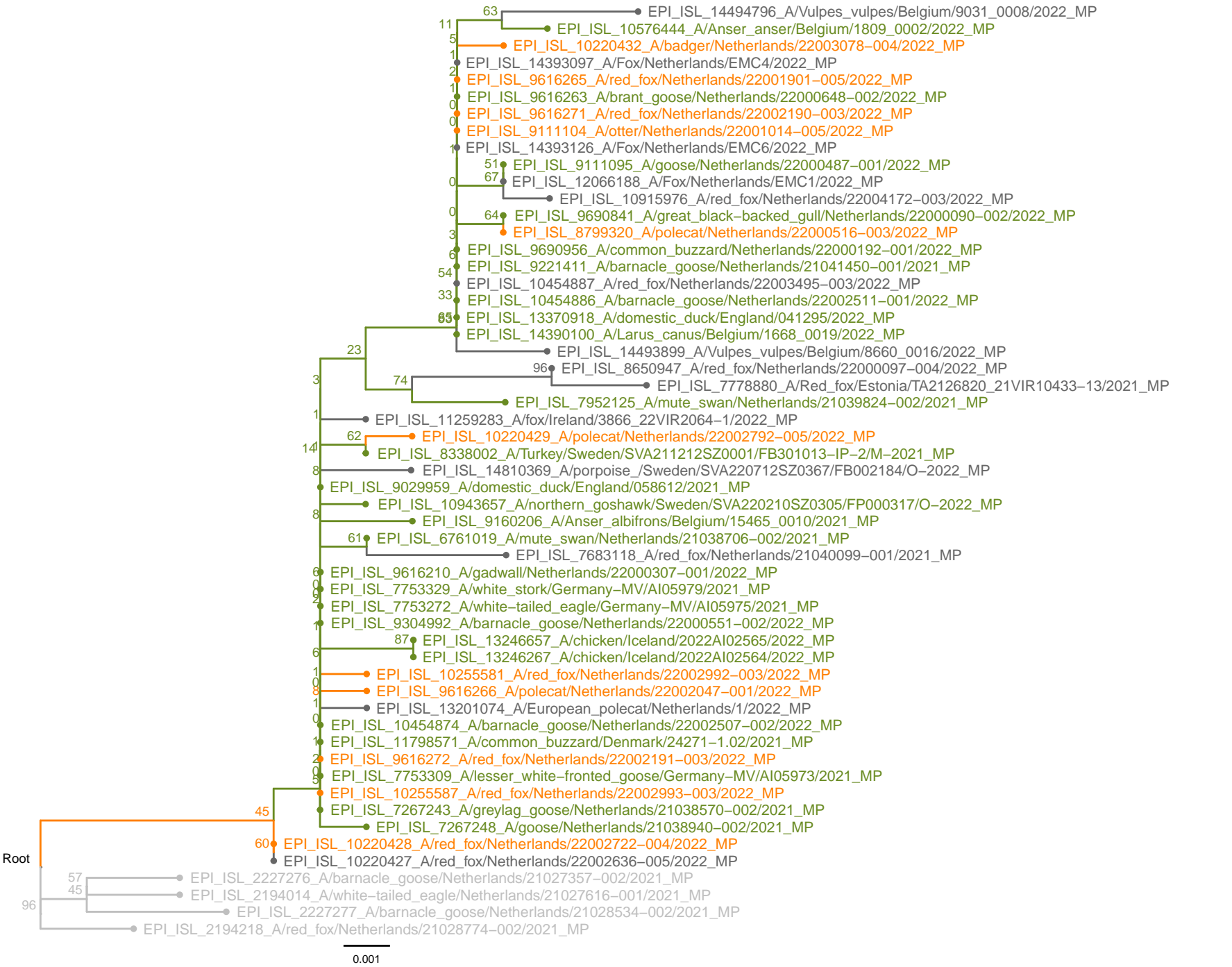

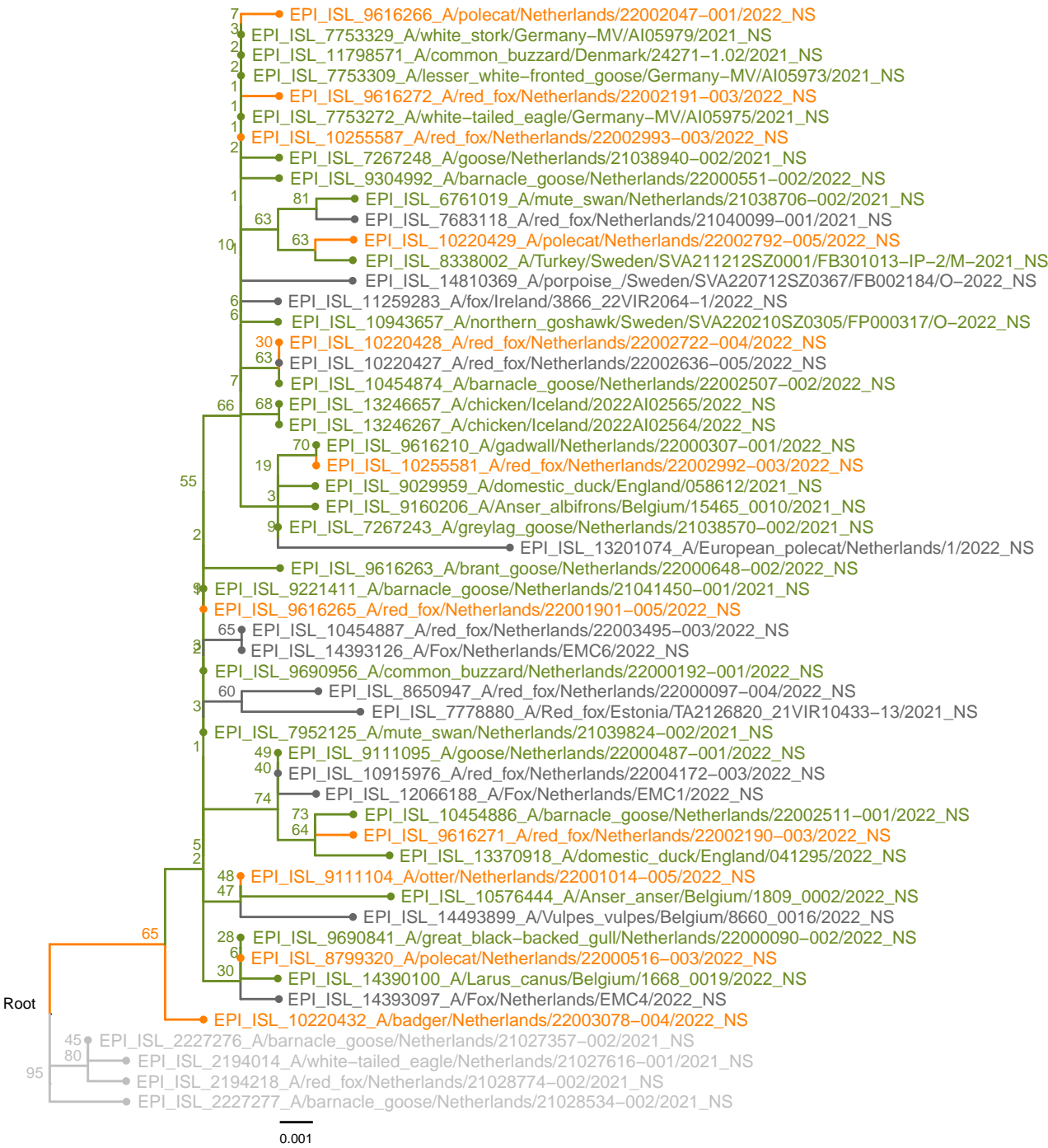

Supplement: Supplementary file 1 [file pathogens-12-00168-s001.zip › Figure S1_ML-trees.pdf]
